# AA amyloidosis with multiorgan involvement secondary to severe hidradenitis suppurativa: A case report

**DOI:** 10.1097/MD.0000000000050113

**Published:** 2026-08-14

**Authors:** Julen Armendariz Gayraud, Francesc Albertí Vich, Isabel Torralba Cloquell, Juan Gabriel Garcias Ladaria, Javier Murillas Angoiti

**Affiliations:** a Department of Internal Medicine, Son Espases University Hospital, Palma de Mallorca, Spain; b Department of Pathology, Son Espases University Hospital, Palma de Mallorca, Spain; c Department of Dermatology, Son Espases University Hospital, Palma de Mallorca, Spain.

**Keywords:** axonal polyneuropathy, case report, hidradenitis suppurativa, nephrotic-range proteinuria, secondary (AA) amyloidosis

## Abstract

**Rationale:**

Secondary (amyloid A [AA]) amyloidosis is a rare but serious systemic complication of chronic inflammatory disorders. Hidradenitis suppurativa (HS) represents an uncommon cause, with approximately 20 cases reported, usually associated with severe, extensive, or long-standing disease.

**Patient concerns:**

A 67-year-old man with severe anogenital HS (Hurley stage III), partially controlled with off-label ustekinumab, developed progressive lower-limb edema, dyspnea, distal limb hypesthesia, and weakness, eventually requiring a wheelchair.

**Diagnoses:**

Laboratory investigations revealed persistently elevated inflammatory markers, severe hypoalbuminemia (16.4 g/L), nephrotic-range proteinuria (14 g/24 hours), and bilateral pleural effusions. Cardiac assessment showed increased left ventricular wall thickness, low QRS voltage on electrocardiography, and preserved systolic function, while neurological studies demonstrated severe axonal sensorimotor polyneuropathy. Renal biopsy showed Congo red-positive deposits with apple-green birefringence and positive immunohistochemical staining for amyloid A, confirming AA amyloidosis.

**Interventions:**

Treatment included systemic corticosteroids, initiation of secukinumab (anti-interleukin-17A), intravenous ertapenem as induction therapy for severe HS, angiotensin-converting enzyme inhibitors, nutritional support, diuretics, and prophylactic anticoagulation.

**Outcomes:**

Despite a decrease in inflammatory markers and a partial reduction in nephrotic-range proteinuria, severe hypoalbuminemia and refractory anasarca persisted. The patient died approximately 5 to 6 weeks after diagnosis because of complications related to advanced systemic disease.

**Lessons:**

This case illustrates that persistent systemic inflammation may occur in patients with severe or refractory HS despite apparent clinical stability of skin lesions. Although AA amyloidosis remains a rare complication, unexplained proteinuria or progressive hypoalbuminemia in selected high-risk patients should prompt consideration of AA amyloidosis to allow timely diagnostic evaluation before irreversible organ damage develops.

## 1. Introduction

Amyloid A (AA) amyloidosis is an uncommon systemic disorder characterized by extracellular deposition of amyloid fibrils derived from circulating serum amyloid A, an acute-phase reactant produced persistently in the setting of chronic inflammation. This form of amyloidosis can involve multiple organs – most notably the kidneys, heart, liver, and peripheral nervous system – leading to progressive functional impairment and potentially irreversible outcomes if not identified and treated promptly.

Historically, chronic infections such as tuberculosis and osteomyelitis were the predominant causes of AA amyloidosis. However, in recent decades, immune-mediated and autoinflammatory conditions – including rheumatoid arthritis, spondyloarthritis, inflammatory bowel disease, and familial Mediterranean fever – have emerged as leading contributors. In this context, hidradenitis suppurativa (HS), although much less common, is a recognized trigger, especially in severe, extensive, or long-standing forms, which carry a significantly increased risk of amyloidosis (odds ratio: 17.5; 95% confidence interval: 3.6–84.4; *P* < .001),^[1]^ with only around 20 cases reported in the literature.^[2]^ HS is a chronic, relapsing, and debilitating inflammatory dermatosis whose sustained systemic inflammatory burden can predispose patients to severe complications such as AA amyloidosis.

Although the association between HS and AA amyloidosis is rare, recognizing this complication is crucial in patients with severe and persistent inflammatory skin disease. The few reported cases typically occur in the context of long disease duration and extensive involvement, making early clinical suspicion challenging. We present the case of a man with refractory HS who developed AA amyloidosis with multiorgan involvement, underscoring the importance of considering this systemic complication in patients with persistently elevated inflammatory markers, even when the cutaneous disease appears clinically stable.

## 2. Case report

### 2.1. Patient information

A 67-year-old man was functionally dependent for most basic activities of daily living due to progressive lower-limb weakness evolving over several months. His past medical history included type 2 diabetes mellitus, chronic iron-deficiency anemia secondary to a perineal fistula with occasional bleeding, benign prostatic hyperplasia, lower-limb lymphedema, and an axonal demyelinating polyneuropathy attributed to prior adalimumab use. He had been followed by Dermatology since 2018 for severe anogenital HS (Hurley stage III) with scrotal lymphedema and a “catastrophic” penis, treated off-label with ustekinumab, which had achieved partial clinical control (Fig. 1). The patient declined surgical intervention due to its highly mutilating nature.

**Figure 1.**
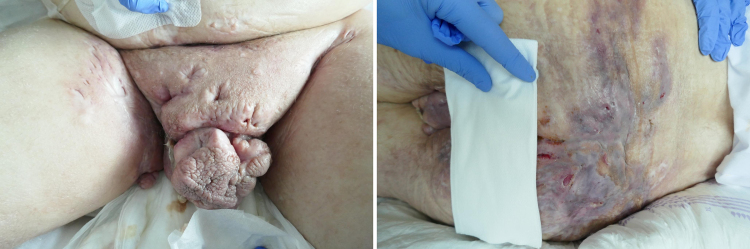
Severe anogenital HS (Hurley stage III). Left: catastrophic penis with multiple retractile scars on the thighs and pubis, along with generalized subcutaneous edema. Right: active inflammatory lesions with ulcers, abscesses, fistulas, and sclerotic plaques in the sacral and gluteal regions. HS = hidradenitis suppurativa.

### 2.2. Clinical findings (presentation)

In late 2024, he was referred to Internal Medicine for progressive lower-limb edema, worsening dyspnea, paresthesias, and bilateral leg weakness, which rendered him unable to walk and dependent on a wheelchair.

### 2.3. Timeline

- 2018: Dermatology follow-up initiated for severe anogenital HS (Hurley stage III) with scrotal lymphedema; off-label ustekinumab started with partial clinical control; surgery declined.
- 2022: Persistently elevated inflammatory markers (C-reactive protein/erythrocyte sedimentation rate [ESR]) and mild hypoalbuminemia documented.
- Late 2024 (outpatient): Progressive edema, dyspnea, paresthesias, and worsening leg weakness (wheelchair dependence); hypoalbuminemia worsened to 16.4 g/L; N-terminal pro-B-type natriuretic peptide > 14,000 pg/mL; pleural effusions; low-voltage electrocardiogram (ECG); left ventricular (LV) wall thickening on echocardiography; ustekinumab discontinued (suspected neurological toxicity); electromyography: severe axonal length-dependent sensorimotor polyneuropathy.
- A few weeks later (admission): Worsening anasarca and HS flare with extension to the trunk/back/lower limbs; nephrotic-range proteinuria (14 g/24 hours) with preserved renal function; albumin <20 g/L; ESR 99 mm/h; bilateral pleural effusions requiring thoracentesis; fat biopsy negative; technetium-99m pyrophosphate scan negative for transthyretin amyloidosis; paraprotein work-up negative.
- During admission: Renal biopsy confirmed AA amyloid deposition (Fig. 2).
- Treatment phase: Corticosteroids + oral antibiotics; temporary reintroduction of ustekinumab, then discontinuation; ertapenem intravenous induction (6 weeks); secukinumab induction, then maintenance; angiotensin-converting enzyme inhibitors; diuretics; nutritional support; prophylactic anticoagulation.

**Figure 2.**
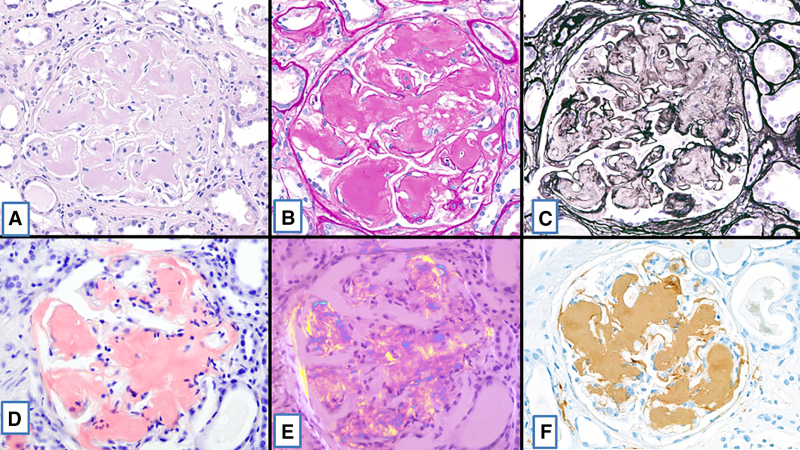
Renal biopsy. Thirteen glomeruli with extensive acellular mesangial deposits appearing pale on hematoxylin-eosin (A), weakly PAS-positive (B), silver-negative (C), Congo red-positive (D) with apple-green birefringence under polarized light (E). Immunohistochemistry was positive for amyloid A (F), negative for kappa, lambda, and TTR (not shown). Amyloid deposition was also noted in vascular walls. PAS = periodic acid–Schiff, TTR = transthyretin.

### 2.4. Diagnostic assessment

Laboratory review showed persistently elevated acute-phase reactants (C-reactive protein and ESR) and mild hypoalbuminemia documented since 2022, initially interpreted as nonspecific but progressively worsening to severely low levels (16.4 g/L) by the time of his outpatient visit. Additional findings included N-terminal pro-B-type natriuretic peptide > 14,000 pg/mL, normocytic anemia, and elevated inflammatory markers. Chest X-ray revealed bilateral pleural effusions; ECG showed low voltage; and echocardiography demonstrated increased LV wall thickness with mild pericardial effusion and frequent ventricular extrasystoles, without systolic dysfunction. Electromyography of the legs revealed severe, axonal, length-dependent sensorimotor polyneuropathy.

A few weeks later, he was admitted for worsening anasarca and a significant exacerbation of skin lesions extending to the trunk, back, and lower limbs. During hospitalization, severe nephrotic-range proteinuria (14 g/24 hours) was confirmed with preserved renal function, persistent hypoalbuminemia < 20 g/L, ESR of 99 mm/h, and moderate-to-severe bilateral pleural effusions requiring thoracentesis. Given this constellation of findings, infiltrative disease (amyloidosis) was suspected. Diagnostic challenges included an initial negative subcutaneous fat biopsy, which required further invasive testing to establish the diagnosis; technetium-99m pyrophosphate scintigraphy showed no cardiac uptake suggestive of transthyretin amyloidosis; and a work-up for paraproteinemia showed no monoclonal component with normal free light-chain ratios. Ultimately, renal biopsy established systemic AA amyloidosis, showing Congo red-positive deposits with apple-green birefringence under polarized light and positive immunohistochemical staining for AA (Fig. 2). Electron microscopy further demonstrated disorganized fibrils measuring 10 to 12 nm in diameter, consistent with amyloid deposition (Fig. 3).

**Figure 3.**
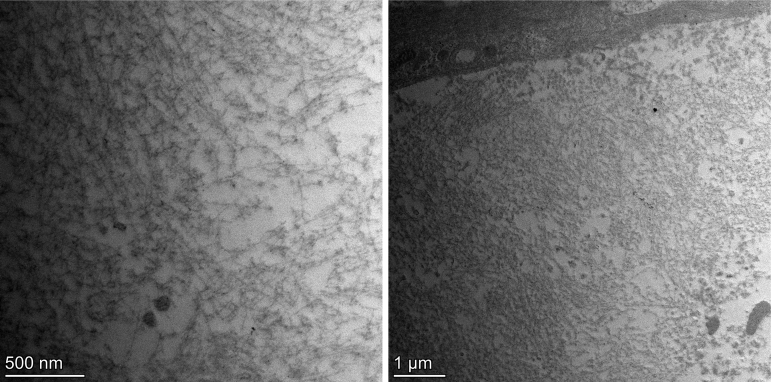
Electron microscopy of renal biopsy. Disorganized fibrils measuring 10 to 12 nm in diameter, typical of amyloidosis.

### 2.5. Therapeutic interventions

During a severe inflammatory flare with new pustular HS lesions, Dermatology was consulted. Initial management included systemic corticosteroids and oral antibiotics, and ustekinumab was reintroduced at the previous dose. However, due to its limited efficacy in this condition and lack of formal indication,^[3]^ it was subsequently discontinued. Use of anti-tumor necrosis factor alpha therapy (adalimumab) was considered but not pursued because of concern about severe heart failure in the setting of suspected cardiac amyloid involvement. Treatment was then initiated with secukinumab (anti-interleukin-17A) at 300 mg weekly for 5 weeks as induction, followed by monthly maintenance dosing. Additionally, a few days prior, intravenous ertapenem had been started for 6 weeks as induction therapy for severe, refractory HS, following literature-supported strategies to rapidly reduce the inflammatory burden and prepare for subsequent antibiotic consolidation.^[4]^ Angiotensin-converting enzyme inhibitors were introduced to reduce proteinuria. The comprehensive management plan also included nutritional support, diuretics, and prophylactic anticoagulation.

### 2.6. Follow-up and outcomes

At the time of diagnosis, the patient presented with advanced multiorgan involvement, including renal manifestations (nephrotic-range proteinuria and severe hypoalbuminemia), cardiac features (low-voltage ECG with increased LV wall thickness), and neurological impairment (severe axonal sensorimotor polyneuropathy), which collectively indicated a poor prognosis. Despite initiation of targeted anti-inflammatory and supportive therapy, the patient’s clinical course was unfavorable. Over the following weeks, inflammatory markers decreased, and a partial reduction in nephrotic-range proteinuria was observed; however, severe hypoalbuminemia persisted. Treatment adherence was ensured during hospitalization, and tolerability was acceptable; nevertheless, the clinical response was limited by advanced multiorgan involvement. The patient developed progressive and refractory anasarca with worsening functional status. Approximately 5 to 6 weeks after the diagnosis of AA amyloidosis, he died due to complications related to advanced systemic disease, despite ongoing medical management.

Patient perspective: *Not available, as the patient died shortly after diagnosis, and no written perspective could be obtained.*

### 2.7. Ethics and consent

Ethics committee approval was not required for this single-patient case report in accordance with institutional policy. Written informed consent was obtained from the patient for publication of the clinical details and accompanying images.

## 3. Commentary

HS is a chronic, relapsing inflammatory dermatosis whose severity does not always correlate with the extent of visible skin involvement, especially in advanced or partially treated stages. Failure to treat or the undertreatment of these inflammatory diseases not only compromises patients’ quality of life and functionality but also carries a risk of mortality due to complications associated with the chronic inflammatory burden, such as AA amyloidosis or HS-associated cutaneous squamous cell carcinoma. In this case, progression to AA amyloidosis with renal, cardiac, and neurological involvement occurred in a patient whose HS was partially controlled with immunomodulatory therapy, achieving apparent clinical stability that delayed diagnostic suspicion.

This case illustrates how an apparently “controlled” chronic inflammatory disease can sustain persistent subclinical inflammation, as evidenced by consistently elevated acute-phase reactants. This ongoing inflammation promotes progressive deposition of AA fibrils derived from serum amyloid A, silently leading to multiorgan AA amyloidosis. In this context, renal involvement – typically presenting as proteinuria – is the earliest and most frequent manifestation, present in up to 95% of patients with secondary AA amyloidosis, and is considered the main prognostic factor.^[5]^ Its detection should be regarded as an early warning sign and an opportunity to intervene before irreversible clinical deterioration occurs.

The lack of correlation between apparent HS control and underlying systemic progression highlights the need for close monitoring in patients with severe or refractory disease. Routine laboratory screening of all patients with HS is not supported by current evidence; however, in selected patients with severe, long-standing, or refractory disease and persistently elevated inflammatory markers, unexplained proteinuria or progressive hypoalbuminemia should prompt consideration of AA amyloidosis and timely diagnostic evaluation, including renal biopsy when clinically indicated. Controlling inflammation remains the most effective strategy to stabilize or even reverse amyloid deposition. Progressive hypoalbuminemia, inflammatory anemia, or indirect signs of cardiac or neurological involvement should prompt active consideration of amyloidosis and should not delay the performance of renal biopsy in unexplained nephrotic syndrome.

Finally, this case underscores the importance of a multidisciplinary approach involving dermatology, internal medicine, neurology, and nephrology to enable early adjustment of immunomodulatory therapy and implementation of targeted supportive measures. Such coordinated management is essential to improve patient outcomes and prevent potentially severe and avoidable complications.

## Author contributions

**Conceptualization:** Julen Armendariz Gayraud.

**Data curation:** Julen Armendariz Gayraud.

**Writing – original draft:** Julen Armendariz Gayraud.

**Writing – review & editing:** Francesc Albertí Vich, Isabel Torralba Cloquell, Juan Gabriel Garcias Ladaria, Javier Murillas Angoiti.
